# Estimating population burdens of occupational disease

**DOI:** 10.5271/sjweh.4007

**Published:** 2022-02-25

**Authors:** David Coggon

**Affiliations:** MRC Lifecourse Epidemiology Centre University of Southampton Southampton General Hospital Southampton, UK Email: dnc@mrc.soton.ac.uk

Knowing the public health impact of occupational hazards is important for prioritization of preventive and mitigating measures and in monitoring how well they succeed. Information is needed on attributable morbidity and mortality, both globally and by national/regional jurisdiction. The best method of estimating population burdens will vary according to the nature of the hazard.

One important consideration is whether health effects can be ascribed to work with confidence in the individual. Such attribution is straightforward where a disease occurs only as a consequence of occupational exposure (eg, coal workers’ pneumoconiosis, byssinosis). Alternatively, a link to occupation can sometimes be established through clinical investigation. For example, allergic contact dermatitis may confidently be attributed to work where it is associated with demonstrable sensitization to an agent encountered only in the workplace; and the role of work in an acute injury or poisoning may be clear from its circumstances and timing. Even where a disorder is not occupational in origin, it may be made worse by exposures in the workplace to an extent that can be determined in the individual case. For example, exacerbation of pre-existing asthma by occupational inhalation of irritants may be apparent from serial measurements of lung function when an employee is at, and away from, work.

In such circumstances, public health burden can be estimated by aggregating data on individual cases, either across the population as a whole, or in a representative subsample. Possible sources of information include routine surveillance schemes such as the Health and Occupation Research (THOR) Network (1), data on claims for industrial injuries compensation (provided they are sufficiently accurate and complete), and ad hoc surveys in representative samples of the population. Where a disease has material fatality (eg, silicosis), counts of deaths may provide a good measure of attributable mortality.

More commonly, occupational disorders are not specific to work, and there is no reliable way of determining occupational contribution in the individual case. The hazard may increase the probability and/or the average severity of a disease. For example, asbestos makes development of lung cancer more likely, while coal mine dust causes chronic obstructive pulmonary disease (COPD) through incremental loss of lung function. Either way, the need is to determine how much morbidity or mortality would be eliminated across the population, if the relevant occupational exposure were removed. To this end, epidemiological data comparing health outcomes in people according to their exposure must be combined with information on the prevalence and distribution of exposure in the population for which an estimate is sought.

This is the approach underpinning the WHO/ILO analysis that is reported in Pega et al’s paper (2). Estimates of relative risk for paired combinations of occupational risk factor and disease were collated with data on the population prevalence of exposure to calculate population attributable fractions (PAF) (3), which then were multiplied by estimates of the total population impact of the disease (in terms of deaths and disability-adjusted life-years) to derive burdens attributable to occupation (2).

The analysis was necessarily restricted to combinations of risk factor and disease for which there was judged to be adequate evidence, but it also has other important limitations, not all of which are acknowledged and discussed. Some of the assumed hazards are questionable. For example, occupational exposure to formaldehyde is estimated to account for some 350–400 deaths per year from leukemia. Although the International Agency for Research on Cancer has classified formaldehyde as a human carcinogen (4), that decision was controversial, and the systematic review and meta-analysis from which the relative risk was derived concluded that “on balance, these data do not provide consistent support for a relationship between formaldehyde exposure and leukemia risk” (5). Similarly, doubts have been cast on the assumed hazard of ischemic heart disease from long working hours, at least among people of higher socioeconomic status (6).

A second problem lies in the ambiguous specification of some risk factors. The analysis attributes large numbers of deaths from COPD to occupational exposure to “particulate matter, gases and fumes” (2). It is unclear, however, what exactly is implied by that term. The many and varied particulates, gases and fumes that people encounter through their work differ widely in their toxicity. If the mix of such pollutants differed between the studies that were used to estimate the prevalence of exposure, and those used to estimate relative risk, then major bias is possible.

Even where risk factors are specified more precisely (eg, sulfuric acid), there are challenging complexities in the characterization and classification of exposure. Impacts at an individual level, whether on risk of disease or its severity, can vary enormously according to the timing, duration and intensity of exposures. An earlier report suggests that for many occupational carcinogens, exposures were classed to three levels (background/low/high) (7). However, within such broad categories, there may be substantial heterogeneity of risk. In the WHO/ILO analysis, many of the risk estimates for occupational carcinogens come from industrial cohort studies, which have tended to focus on working populations known or expected to have relatively high intensity and duration of exposure (making any risks more readily detectable). In contrast, data on the prevalence of exposure derive from studies that aimed to ascertain the full extent of exposure in the population, even if only at a modest level. Within a broad exposure category, a meta-estimate of risk from published cohort studies may not be applicable to the distribution of exposures within that category in the general population, and such incompatibility could in some cases cause population burden to be seriously overestimated.

Another challenge when extrapolating risk estimates from samples to populations is the potential for effect modification. The burden of back and neck pain was assessed in relation to “occupational exposure to ergonomic factors” (2), defined as “proportion of the population who are exposed to ergonomic risk factors for low back pain at work or through their occupation” (3). However, it is unclear how well the calculation allowed for major variation between countries in the individual risk of musculoskeletal pain and disability from specified occupational activities (8, 9).

One way round these problems is to estimate population burden more directly, using the same study to provide information both on the distribution of exposure in the population and the effects of that exposure. For example, a national analysis of mortality by occupation has been used to estimate excess deaths from COPD among coal miners in England and Wales (10). While such analyses have other important limitations (for example, mortality as recorded on death certificates is an imperfect marker for disease, and full account cannot be taken of changes in occupation over a lifetime), the risk estimates that they generate are an average for exposure as it occurs in the population as a whole. In this respect, provided the ascertainment of exposure is reasonably sensitive (specificity is less critical), estimates of population burden will be less prone to bias.

Given the many sources of uncertainty in the WHO/ILO analysis, only some of which have been highlighted here, it is surprising that such tight 95% uncertainty ranges are reported. For example, the uncertainty in thousands of deaths globally in 2017 from occupational exposure to formaldehyde is reported as 1 to 1, and that for lung cancer from occupational exposure to diesel exhaust as 16 to 20 (3). This is a concern because findings published under the auspices of authoritative international bodies such as WHO and ILO are liable to be accepted by many without question.

As occupational health researchers and practitioners, we are naturally disposed to champion the importance of health protection in the workplace, but that enthusiasm should not compromise scientific rigor. Findings that appear to support our case must be scrutinized with the same care as those that call it into question. When estimating population burdens of disease from occupational hazards, we should aim if possible to triangulate between different analytical approaches and sources of data, carefully considering and acknowledging sources of uncertainty. The potential for error will often be greatest for exposures that are relatively prevalent in the working population (eg, long working hours), for which small differences in excess relative risk can translate into substantial differences in estimated burdens of disease at population level.
